# Identification of biomarkers of shrinkage modes after neoadjuvant therapy in HER-2 positive breast cancer

**DOI:** 10.1097/JS9.0000000000002349

**Published:** 2025-03-26

**Authors:** Zhao Bi, Yue Zhang, Xian-Rang Song, Wen-Hao Zheng, Peng Chen, Peng-Fei Qiu, Yan-Bing Liu, Yong-Jin Lu, Xing-Guo Song, Yong-Sheng Wang

**Affiliations:** a Shandong Cancer Hospital and Institute, Shandong First Medical University and Shandong Academy of Medical Sciences, Jinan, Shandong, People’s Republic of China; b Shanghai Pudong New Area Center for Disease Control and Prevention, Shanghai, People’s Republic of China; c Rizhao Central Hospital, Rizhao, Shandong, People’s Republic of China

**Keywords:** breast cancer, neoadjuvant therapy, nomogram, RUVBL1-AS1, shrinkage modes

## Abstract

**Purpose:**

A nomogram to predict shrinkage modes after neoadjuvant therapy (NAT) was constructed in HER-2 positive (HER2+) breast cancer. The value and mechanism of targeting long noncoding RNA (lncRNA) as efficacy prediction biomarker was also evaluated.

**Methods:**

All enrolled patients received six cycles of chemotherapy (Docetaxel + Carboplatin) and anti-HER-2 dual-targeted therapy (Trastuzumab + Pertuzumab) before surgery. According to pathological three-dimensional (3D) models of residual tumor from 71 HER2+ patients, shrinkage modes were divided into concentric shrinkage mode (CSM) and non-CSM (NCSM). LncRNAs in core biopsy tissues in the CSM and NCSM groups were selected by microarray and validated by RT-PCR. A nomogram was constructed to predict shrinkage modes after NAT in combination with clinical-pathological and transcriptome signatures. Cell proliferation was used CCK-8 and colony formation assay. PAPIS Kit was used to perform nuclear and cytoplasmic separation. The cell drug resistance assays were to explore the value of paclitaxel. The ChIRP-MS assay was to search RNA-binding proteins and verified by WB. Cell cycle analysis was carried out by flow cytometry.

**Results:**

Independent predictors of NCSM were lymph nodes downstaging after NAT, mammographic malignant calcification, hormone receptor expression, and RUVBL1-AS1 expression. A nomogram was constructed in combination with these predictors, which showed an area under the curve of 0.883, supporting the predictive power of the method. Overexpression of RUVBL1-AS1 inhibited HER2+ cells proliferation. Overexpression of RUVBL1-AS1 increased the number of cells in G1/S phase and decreased that of cells in G2 phase. RUVBL1-AS1 increased paclitaxel resistance and downregulated VCP expression. RUVBL1-AS1 affects cell cycle progression by downregulating VCP, resulting in the reduction of cells in G2/M phase, thereby weakening the sensitivity to paclitaxel.

**Conclusion:**

The nomogram could accurately predict shrinkage modes after NAT, and may help guide the individualized selection of breast conserving surgery candidates after NAT. RUVBL1-AS1 might be a promising therapeutic target of paclitaxel-based chemotherapy inHER2+ breast cancer.

## Introduction

**Highlights**

- Accurately predict shrinkage modes could guide breast-conserving surgery after neoadjuvant therapy.
- The nomogram indicated that patients with large primary tumor, mammographic malignant calcification, positive hormone receptor expression, high expression RUVBL1-AS1 were more likely to present with nonconcentric shrinkage mode.
- RUVBL1-AS1 affects cell cycle progression by downregulating VCP, resulting in the reduction of cells in G2/M phase, thereby weakening the sensitivity to paclitaxel in HER-2 positive breast cancer.

**Figure 1.**
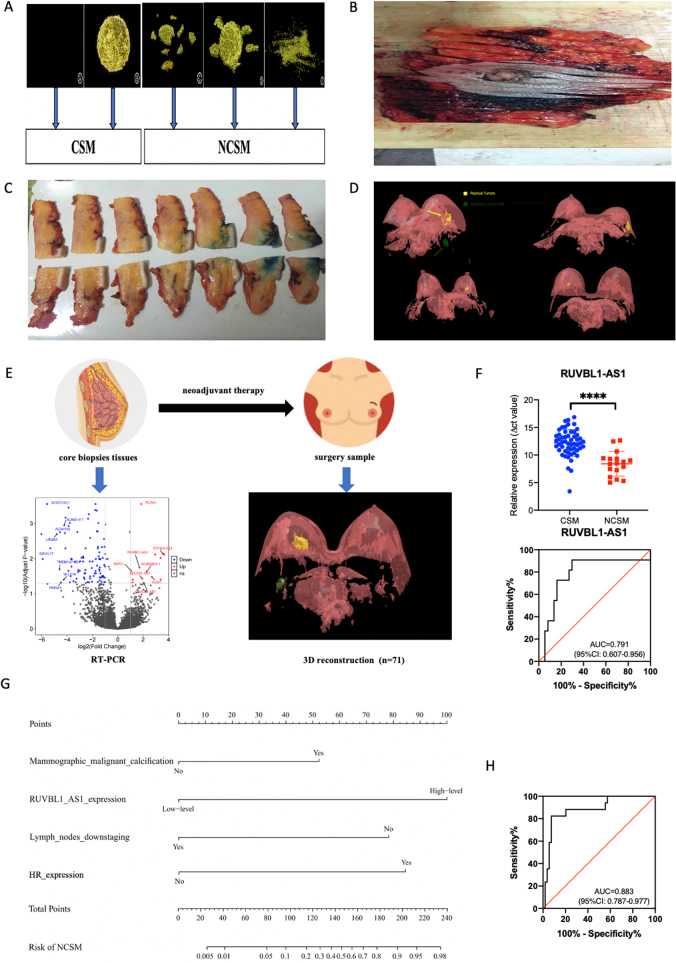
The shrinkage modes of residual tumors after NAT ^[^10^]^. (A) The shrinkage modes. The surgical pCR and solitary lesion without surrounding lesions were divided into CSM. Other shrinkage modes were divided into NCSM. (B) The specimen was cut into several blocks at 5-mm intervals based on the markers. (C) The extent of residual tumors was delineated under microscope. (D) The pathology 3D reconstruction model of residual tumor after NAT. (E) The consort diagram of the study. (F) The different expression and AUC of RUVBL1-AS1 in predicting NCSM after NAT. (G) The nomogram of predicting NCSM after NAT. (H) The ROC curve of the nomogram.

HER-2 positive (HER2+) breast cancer has a high degree of malignancy and poor prognosis and is associated with a poor prognosis^[^1^]^. Neoadjuvant therapy (NAT) is the main treatment for patients with HER2+ breast cancer^[^2,3^]^. Treatment guidelines of the NAT protocol for HER2+ breast cancer recommend a combination of target therapy with chemotherapy. In addition, the efficacy of other regimens, including Pyrotinib, Trastuzumab Deruxtecan, and Atezolizumab, has also been studied^[^4–8^]^. Although these regimens have yielded good results, dual-target therapy (Trastuzumab + Pertuzumab) remains the main protocol in the neoadjuvant setting^[^9^]^. One important clinical benefit of NAT is that it can downstage the tumor. As a result, inoperable tumors may become operable and patients with large tumors can undergo breast-conserving surgery (BCS) to facilitate better cosmetic outcomes^[^10^]^. For patients who will undergo BCS after NAT, accurate determination of shrinkage modes after NAT is important to ensure negative margins. In previous study, we constructed MRI and pathological three-dimensional (3D) models of the residual tumor and defined the shrinkage modes, which were oriented by BCS purpose after NAT. These modes include concentric shrinkage modes (CSM) and non-CSM (NCSM) (Fig. 1A)^[^10^]^. We confirmed that identifying shrinkage modes can help guide the individualized selection of BCS candidates and resection scope after NAT. The guidelines and expert consensus recommend that patients with CSM are suitable for BCS after NAT.

The shape of residual tumor after NAT is usually assessed using magnetic resonance imaging (MRI); however, this method is prone to some error in the assessment of shrinkage modes after NAT^[^11,12^]^. Therefore, it is critical to identify biomarkers capable of accurately predicting shrinkage modes. Long noncoding RNA (lncRNA) belongs to a category of RNA with over 200 nucleotides in length and lacks protein-coding ability, and it is characterized by poor sequence conservation and complexity of regulatory mechanism. Recent studies have highlighted the potential value of lncRNA as predictive biomarkers^[^13,14^]^. However, there are few studies exploring the potential of lncRNA for predicting shrinkage modes after NAT.

Because HER2+ patients account for 60% of the population suitable for NAT, so this study focused on HER2+ breast cancer to benefit a larger population of patients. The purpose of this study was to construct a nomogram for predicting shrinkage modes after NAT in patients with HER2+ breast cancer in combination with clinical-pathological and transcriptome signatures. In addition, we explored the value and mechanism of target lncRNA as efficacy prediction biomarker.

## Methods

### Patients

Between April 2018 to 2020, 71 patients treated at our Hospital Breast Cancer Center were enrolled in this study. Adult women were included in this study if they (1) had histologically confirmed HER2+ breast carcinoma; (2) were clinical staging T_2–4_N_1–3_M_0_; (3) agreed to undergone MRI and pathological 3D reconstruction after NAT. Patients were excluded according to the pre-established exclusion criteria if they had undergone therapy prior to NAT, concurrent cancer, bilateral breast cancer, or distant metastases.

### Treatment

Before NAT, core biopsies of primary tumor were taken guided by ultrasound. All patients in this study received six cycles of chemotherapy (Docetaxel + Carboplatin) and anti-HER-2 dual-targeted therapy (Trastuzumab + Pertuzumab) before surgery. Patients received adjuvant therapy according to the postoperative pathological status, in accordance with the guidelines and expert consensus at the time. hormone receptor (HR) positive patient received endocrine therapy. Patients who did not reach pathological complete response (pCR) received T-DM1 intensive therapy, and those who reached pCR would receive dual-targeted therapy (Trastuzumab + Pertuzumab).

### Serial sections of breast specimens and pathological 3D reconstruction

After surgery, the specimens were cut into several blocks at 5 mm intervals (Fig. 1B). Then we made one section of 4–6 μm thick in each block. The sections were cut using a Leica RM2010 slicer (Leica Biosystems, Nussloch, Germany) and stained with hematoxylin and eosin^[^10^]^. Invasive tumors were delineated under microscope, respectively (Fig. 1C). The pathology 3D reconstruction model of residual tumor was presented using 3D-DOCTOR software (Fig. 1D). According to 3D reconstruction model, the shape of residual tumor after NAT included five categories: surgical pCR, solitary lesion without surrounding lesions, multinodular lesions, solitary lesion with adjacent spotty lesions and diffuse lesions. The surgical pCR and solitary lesion without surrounding lesions were divided into CSM. Other shrinkage modes were divided into NCSM.

### LncRNA microarray and validation

LncRNAs in core biopsies tissues between CSM and NCSM groups were selected by microarray. In the previous study, the Arraystar human lncRNA microarray V5.0 was designed for the profiling of human lncRNA^[^14^]^. Finally, about 19 307 lncRNAs can be detected by lncRNA microarray. We validated the different expression of lncRNAs by RT-PCR. The qPCR primers are listed in Supplemental Digital Content Table 1, available at: http://links.lww.com/JS9/E29. The association of different clinical-pathological variables and lncRNAs expression level with shrinkage modes was analyzed. A nomogram was developed based on variables in the final model with *p* < 0.05 using “rms” package for R.

### The exploration of mechanism of target lncRNA

Then we used human HER2+ breast cancer cell lines (BT-474 and SK-BR-3 cell) to explore the value of RUVBL1-AS1. We explored the cell proliferation using the Cell Counting Kit-8 (CCK-8) and colony formation assay. PAPIS Kit (Life Technologies, USA) was used to perform nuclear and cytoplasmic separation. The cell drug resistance assays were performed to explore the value of paclitaxel and Trastuzumab. The comprehensive identification of RNA-binding proteins by mass spectrometry (ChIRP-MS) assay was performed to search the RNA-binding proteins. The binding proteins verified *via* MS and western blot (WB). Cell cycle analysis was carried out by flow cytometry^[^15^]^.

### Statistical analysis

Statistical analyses were carried out using SPSS Statistics 22.0 software (IBM Corporation, Armonk, NY, USA), R version 3.3.3 software (The R Foundation for Statistical Computing, Austria, Vienna), and GraphPad Prism version 9.0 (GraphPad Software, San Diego, CA, USA). Pearson chi-square test or Fisher exact test was used to perform logistic regression analysis on categorical variables. A nomogram was developed based on variables in the final model with *p* < 0.05 using “rms” package for R. The discrimination of the model was evaluated using the area under the curve (AUC) value of the receiver operating characteristic (ROC) curve. A *p* < 0.05 was considered statistically significant.

## Results

### Patients’ characteristics

The consort diagram of the study was illustrated in Fig. 1E and Supplemental Digital Content Figure 1A, available at: http://links.lww.com/JS9/E26. From April 2018 to 2020, 103 patients were assessed for eligibility, among whom 8 patients were unqualified for eligibility criteria and 20 patients disagreed to undergo pathological 3D reconstruction. There were 75 patients enrolled and received NAT. During the treatment, there were 4 patients were discontinued due to excessive adverse reactions. Finally, 71 patients received NAT and underwent surgery in the final analysis. There were 54 cases in CSM group and 17 cases in NCSM group. The median age of patients was 49 years old (range 25–70 years). The clinical characteristics of patients are summarized in Supplemental Digital Content Table 2, available at: http://links.lww.com/JS9/E30.

### The nomogram of shrinkage modes after NAT

According to the lncRNA microarray data, we screened 48 up-regulated genes and 42 down-regulated genes in NCSM cases. We analyzed the differential expression of above selected lncRNA by RT-PCR between CSM group and NCSM group. The expression of POTEH-AS1, lncRNA-AC009975.1, and RUVBL1-AS1 were elevated significantly in NCSM group compared with in CSM group (*p* value was 0.0038, 0.0017, and 0.0001, respectively) (Fig. 1F, Supplemental Digital Content Figure 1B, C, available at: http://links.lww.com/JS9/E26). The AUC value was 0.730, 0.743, and 0.791, respectively (Supplemental Digital Content Figure 1B, C, available at: http://links.lww.com/JS9/E26). Next, we combined with clinical-pathological and transcriptome signatures to analyze the influence factors of shrinkage modes after NAT.

According to the multivariate logistic analysis, there was significant difference between shrinkage modes and lymph nodes downstaging after NAT (odds ratio (OR) = 20.490, 95% confidence interval (CI): 1.485–282.755, *p* = 0.024), mammographic malignant calcification (OR = 7.606, 95% CI: 1.243–46.528, *p* = 0.028), HR expression (OR = 9.341, 95% CI: 1.512–54.468, *p* = 0.031) and RUVBL1-AS1 expression (OR = 72.369, 95% CI: 3.828–138.075, *p* = 0.004) (Supplemental Digital Content Table 3, available at: http://links.lww.com/JS9/E31). Based on the aforementioned multivariate analysis results, we built the nomogram to predict patients with NCSM (Fig. 1G). The nomogram was internally validated using the bootstrap method, with an AUC of 0.883 (95% CI: 0.797–0.977), indicating that the nomogram had potentially promising predictive power (Fig. 1H).

Therefore, considering the good predictive performance of RUVBL1-AS1, we selected RUVBL1-AS1 as the target gene. RUVBL1-AS1 is located on chromosome 3, with 588 base pairs in length. Then we further explore the biological function of RUVBL1-AS1.

### The role of RUVBL1-AS1 in HER2+ breast cancer cells

**Figure 2.**
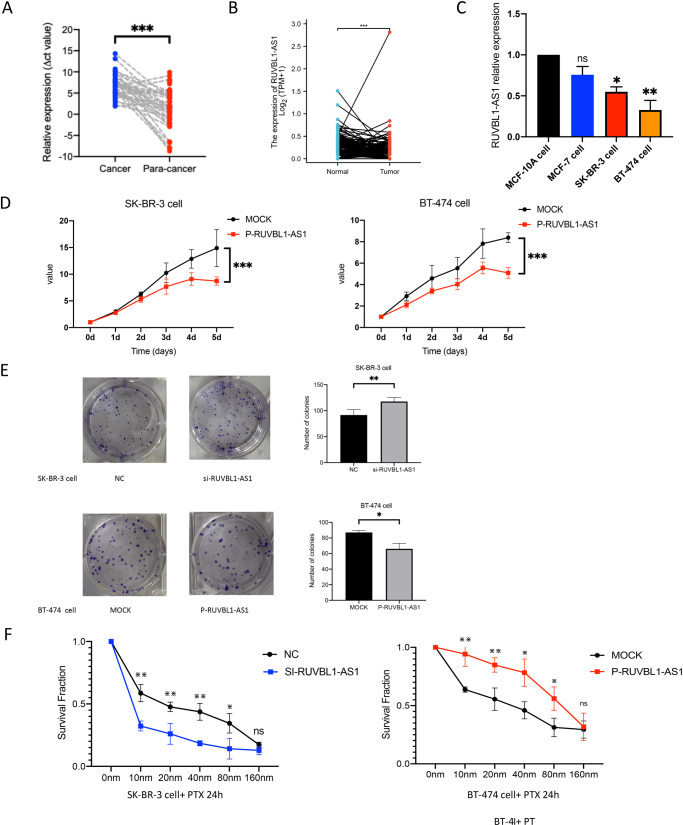
RUVBL1-AS1 inhibits breast cancer cell proliferation. (A) RUVBL1-AS1 was low expression in tumor tissues. (B) TCGA database showed the same result. (C) The expression level of RUVBL1-AS1 in different cells. (D) The overexpression of RUVBL1-AS1 inhibited the growth of HER2+ cells. (E) Representative images of the cloning formation assay showed that overexpression of RUVBL1-AS1 inhibited the growth of HER2+ cells. (F) The growth rate of xenograft was slower, and the weight and volume decreased in P-RUVBL1-AS1 compared with the control.

RUVBL1-AS1 was lower expressed in HER2+ tumor tissues than in their adjacent non-tumor tissues (Fig. 2A). TCGA database showed the same result (Fig. 2B). As shown in Figure 2C, compared to MCF-10A cells, RUVBL1-AS1 was remarkably downregulated in HER2+ breast cancer cell lines (BT-474, and SK-BR-3 cell lines, *p* < 0.05).

Next, we assessed the effects of RUVBL1-AS1 on the growth of HER2+ cells. The transfection efficiency of P-RUVBL1-AS1 and sh-RUVBL1-AS1 are shown in Supplemental Digital Content Figure 1D, available at: http://links.lww.com/JS9/E26. According to CCK-8 assay results, overexpression of RUVBL1-AS1 significantly inhibited the growth and colony formation of BT-474 and SK-BR-3 cells (Fig. 2D, E). Then we explored whether RUVBL1-AS1 inhibits the drug-sensitivity of paclitaxel. The results showed that paclitaxel inhibits HER2+ cell growth in a dose-dependent manner. Overexpression of RUVBL1-AS1 could restrain the drug-sensitivity of paclitaxel in HER2+ cells (Fig. 2F). However, we did not find RUVBL1-AS1 could affect the drug-sensitivity of Trastuzumab in HER2+ cell (Supplemental Digital Content Figure 1E, available at: http://links.lww.com/JS9/E26).

**Figure 3.**
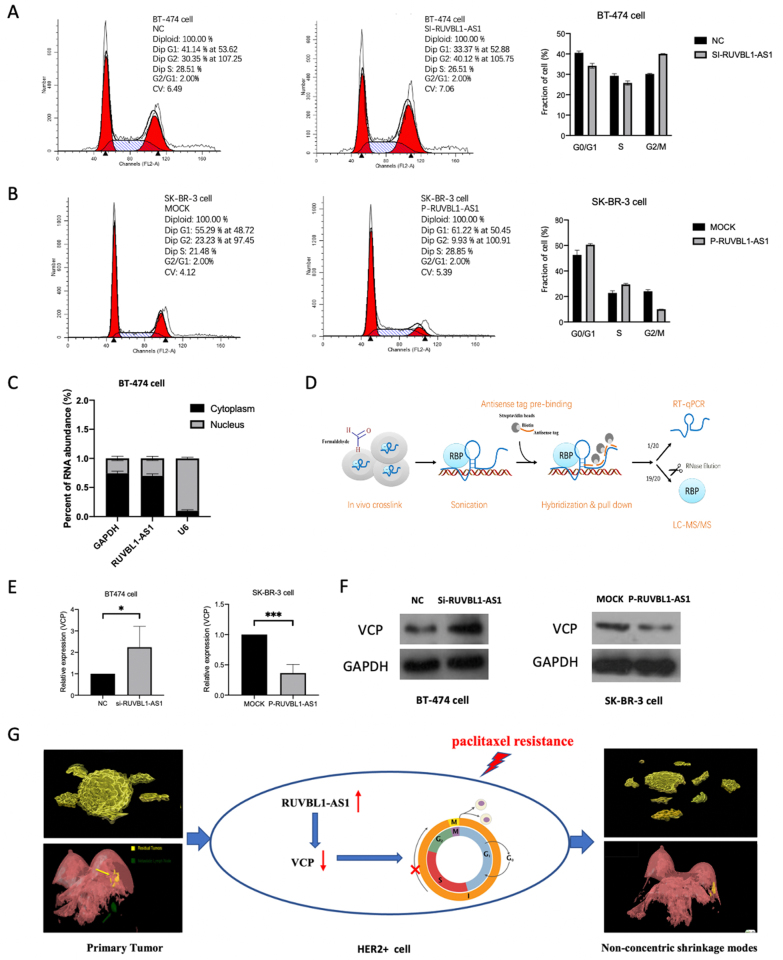
The exploration of potential mechanism of RUVBL1-AS1. (A) Knockdown of RUVBL1-AS1 significantly decreased the proportion of cells in G1/S phases and increased G2/M phases. (B) Overexpression of RUVBL1-AS1 significantly increased the proportion of cells in G1/S phases and decreased G2/M phases. (C) The nuclear-cytoplasmic fractionation assay of RUVBL1-AS1. (D) Schematic of the ChIRP-MS assays. (E) Knockdown of RUVBL1-AS1 could up-regulate the mRNA levels of VCP in HER2+ cells, while overexpression of RUVBL1-AS1 showed the opposite effect. (F) WB analyses showed that knockdown of RUVBL1-AS1 increases the protein levels of VCP, while overexpression of RUVBL1-AS1 showed the opposite effect. (G) The mechanism diagram of this study.

Combined with free tubulin, the paclitaxel promotes the formation of stable microtubules, thereby inhibiting the normal formation of spindle. So, we hypothesized that RUVBL1-AS1 was associated with cell cycle progress. Flow cytometry analysis showed that overexpression of RUVBL1-AS1 significantly increased the proportion of cells in G0/G1 phases and decreased in G2/M phases of HER2+ cells. In contrast, the silencing of RUVBL1-AS1 expression had the opposite effects (Fig. 3A, B). Taken together, these data indicated that RUVBL1-AS1 is required for G0/G1 to G2/M phase transition of HER2+ breast cancer cells.

### RUVBL1-AS1 might regulate cell cycle through modulated VCP expression

To investigate the mechanism of RUVBL1-AS1, we detected the localization of RUVBL1-AS1 in cells. The nuclear-cytoplasmic fractionation assay showed that RUVBL1-AS1 was mainly located within the cytoplasm in HER2+ cells (Fig. 3C). Next, we analyzed the differentially expressed miRNAs after RUVBL1-AS1 overexpression by miRNA sequencing. The results showed that RUVBL1-AS1 overexpression can indeed significantly increase the expression of miRNA-1912-5p and miRNA-196a (Supplemental Digital Content Figure 2A, available at: http://links.lww.com/JS9/E27).

Then, we performed ChIRP-MS assay. Subsequent analysis was based on the quantitative information shown in Figure 3D. Compared with the control group, we identified 28 proteins bound by RUVBL1-AS1. We mainly focused on the top 10 proteins in the iBAQ score (Supplemental Digital Content Figure 2B, available at: http://links.lww.com/JS9/E27). As verified by RT-PCR, the mRNA level of VCP was up-regulated during the knockdown of RUVBL1-AS1 (Fig. 3E). Moreover, WB results showed that up-regulation of RUVBL1-AS1 could decrease the protein levels of VCP in HER2+ cells (Fig. 3F). The above results indicated that RUVBL1-AS1 might regulate cell cycle through modulated VCP expression, thereby inhibiting the drug-sensitivity of paclitaxel. Finally, the breast tumor would shrinkage into NCSM (Fig. 3G). Then we explore the value of VCP (Supplemental Digital Content Figure 2C–E, available at: http://links.lww.com/JS9/E27). Finally, we performed rescue experiments in the Supplemental Digital Content Figure 3, available at: http://links.lww.com/JS9/E28.

## Discussion

In this study, we constructed a nomogram to predict shrinkage modes after NAT in HER2+ breast cancer in combination with clinical-pathological and transcriptome signatures. The nomogram indicated that patients with a large primary tumor, mammographic malignant calcification, HR positive tumors, and high RUVBL1-AS1 expression were more likely to present with NCSM. In addition, we explored the value and potential mechanism underlying the role of RUVBL1-AS1 as a predictive biomarker of shrinkage modes. The results indicated that RUVBL1-AS1 might regulate cell cycle by modulating VCP expression, thereby decreasing the sensitivity to paclitaxel in HER2+ breast cancer. In this study, all patients received docetaxel chemotherapy regimen. Unfortunately, we did not find that RUVB L1-AS1 was associated with targeted therapy resistance. Therefore, RUVBL1-AS1 might be a promising therapeutic target for paclitaxel-based chemotherapy in HER2+ breast cancer.

Assessment of tumor extent can be difficult after NAT, and shrinkage modes may be heterogeneous; surgery is thus technically more difficult than without NAT. The 5-year local-regional reference (LRR) rate can be higher with than without NAT. In patients planning to undergo BCS after NAT, it is important to accurately assess residual tumor extent and shrinkage modes to ensure negative margins and reduce the LRR rate^[^16^]^. The strength of our study is that the nomogram can help guide the individualized selection of BCS candidates and scope of resection after NAT. In addition, it can contribute to decreasing the negative margins distance while maintaining the natural breast shape to improve cosmetic outcomes. In patients with a high likelihood of NCSM, BCS is not recommended because of the need to achieve negative margins. These patients need to be cautious if choosing BCS, at the same time, they also need a more “generous” resection extent.

The correlation between HER-2 status and shrinkage modes may reflect the biological characteristics of the tumors. One possible reason might be the growth characteristic of HER-2 negative subtypes, in which tumor cells tend to grow slowly with a low rate of apoptosis and genetic instability^[^11^]^. Furthermore, tumor cells in these subtypes may be more resistant to preoperative therapy. However, tumor cells in HER2+ subtypes show poor differentiation and a strong proliferation ability, and aggressive tumor cells were more sensitive to therapy. Consistently, Heacock *et al*^[^17^]^ also reported that HER2+ patients are more likely to achieve CSM after neoadjuvant dual-targeted therapy.

The main anti-tumor mechanism of paclitaxel is that it can combine with free tubulin, which promotes the formation of stable microtubules, thereby inhibiting the normal formation of spindle^[^18^]^. In this study, RUVBL1-AS1 may cause paclitaxel resistance by regulating VCP and interfering with cell cycle progression. In the previous study, knockdown of the lncRNA ELF3-AS1 significantly downregulates the expression of miR-33a, miR-33b, and miR-203a, and significantly up-regulation of SNAI2 mRNA and protein, indicating that lncRNA can negatively regulate mRNA and protein expression via miRNA but independent of ceRNA^[^19^]^. In our study, RUVBL1-AS1 overexpression upregulates miRNA-1912-5p and miRNA-196a. So, we hypothesized that the interaction between RUVBL1-AS1 and VCP could provide a negative feedback signal to up-regulate RUVBL1-AS1 expression, which in turn upregulates miRNA-1912-5p and miRNA-196a targeting VCP, resulting in the downregulation of VCP and a decrease in, cells in G2/M phase. As a result, the cell cycle is blocked in G2/M phase, thus effectively preventing the proliferation of cancer cells. In addition, the effect of paclitaxel may be weakened when the proportion of cells in G2/M phase is reduced. These results indicated that RUVBL1-AS1 might be a promising therapeutic target for paclitaxel-based chemotherapy regimen.

This study had several limitations, the most important of which was the small sample size. The lack of external data to verify the accuracy of the nomogram was another limitation. Regulatory mechanisms of RUVBL1-AS1 remain partially understood and will be further examined. Although our study indicated that shrinkage modes can guide BCS practice, the concept has not yet entered into the clinical practice. At the same time, considering the acceptability of BCS in our country and patients’ willingness, only 23.9% of patients finally decided to undergo BCS after NAT. Finally, all patients received docetaxel chemotherapy regimen in our study. Unfortunately, we did not find that RUVB L1-AS1 was associated with targeted therapy resistance. So, in this study, we can only confirm that RUVBL1-AS1 predicts shrinkage modes after paclitaxel-based NAT.

In conclusion, we constructed a nomogram capable of predicting shrinkage modes after NAT when combined with clinical-pathological and transcriptome signatures. This could help guide the individualized selection of BCS candidates after NAT. RUVBL1-AS1 might be a promising predictive biomarker of shrinkage modes after paclitaxel-based NAT in HER2+ breast cancer.

## Data Availability

Not applicable.
